# Familial hypertrophic obstructive cardiomyopathy with the GLA E66Q mutation and zebra body

**DOI:** 10.1186/s12872-016-0262-y

**Published:** 2016-05-10

**Authors:** Masayoshi Oikawa, Nobuo Sakamoto, Atsushi Kobayashi, Satoshi Suzuki, Akiomi Yoshihisa, Takayoshi Yamaki, Kazuhiko Nakazato, Hitoshi Suzuki, Shu-ichi Saitoh, Yuichirou Kiko, Hajime Nakano, Takeharu Hayashi, Akinori Kimura, Yasuchika Takeishi

**Affiliations:** Department of Cardiology and Hematology, Fukushima Medical University, 1 Hikarigaoka, Fukushima, 960-1295 Japan; Department of Diagnostic Pathology, Fukushima Medical University, 1 Hikarigaoka, Fukushima, 960-1295 Japan; Department of Dermatology, Hirosaki University Graduate School of Medicine, 5 Zaifu-cho, Hirosaki, 036-8562 Japan; Department of Molecular Pathogenesis, Medical Research Institute, Tokyo Medical and Dental University, 1-5-45 Yushima, Bunkyo-ku, Tokyo, 113-8510 Japan

**Keywords:** Fabry disease, E66Q mutation, Left ventricular outflow obstruction, Hypertrophic cardiomyopathy

## Abstract

**Background:**

Fabry disease is caused by mutations in the α-galactosidase A (GLA) gene, which is located in X-chromosome coding for the lysosomal enzyme of GLA. Among many gene mutations, E66Q mutation is under discussion for its pathogenicity because there is no clinical report showing pathological evidence of Fabry disease with E66Q mutation.

**Case presentation:**

A 65-year-old Japanese female was referred to our hospital for chest discomfort on effort. Transthoracic echocardiography showed severe left ventricular (LV) hypertrophy with LV outflow obstruction. Maximum LV outflow pressure gradient was 87 mmHg, and Valsalva maneuver increased the pressure gradient up to 98 mmHg. According to medical interview, one of her younger sister and a nephew died suddenly at age 42 and 36, respectively. Another younger sister also presented LV hypertrophy with outflow obstruction. Maximum LV outflow pressure gradient was 100 mmHg, and the E66Q mutation was detected similar to the case. Endomyocardial biopsy specimens presented vacuolation of cardiomyocytes, in which zebra bodies were detected by electron microscopic examination. Although the enzymatic activity of GLA was within normal range, the c. 196G>C nucleotide change, which lead to the E66Q mutation of GLA gene, was detected. We initially diagnosed her as cardiac Fabry disease based on the findings of zebra body. However, immunostaining showed few deposition of globotriaosylceramide in left ventricular myocardium, and gene mutations in the disease genes for hypertrophic cardiomyopathy (HCM), MYBPC3 and MYH6, were detected. Although the pathogenicity of the E66Q mutation cannot be ruled out, hypertrophic obstructive cardiomyopathy (HOCM) was more reasonable to explain the pathophysiology in the case.

**Conclusions:**

This is the confusable case of HOCM with Fabry disease with the GLA E66Q mutation. We have to take into consideration the possibility that some patients with the E66Q mutation may have similar histological findings of Fabry disease, and should be examed the possibility for harboring gene mutations associated with HCM.

## Background

Fabry disease is caused by mutations in the α-galactosidase A (GLA) gene, which is located in X-chromosome coding for the lysosomal enzyme of GLA. To date, more than 500 gene mutations have been identified [1], and clinical manifestations are varied based on the location of the mutation and gender. Among many gene mutations, E66Q mutation is relatively common, especially in Asian people [2]. The E66Q mutation is thought to be a functional single-nucleotide polymorphism and not a disease-causing mutation, because residual enzymatic activity is relatively preserved and the mutation site is distant from the enzymatic active site [3]. Histological examination is helpful to diagnose Fabry disease, and zebra body is the strong evidence to suggest an accumulation of globotriaosylceramide (Gb3). In the present paper, we demonstrated confusable case of hypertrophic obstructive cardiomyopathy (HOCM) with cardiac Fabry disease associated with the E66Q mutation.

## Case presentation

**Fig. 1:**
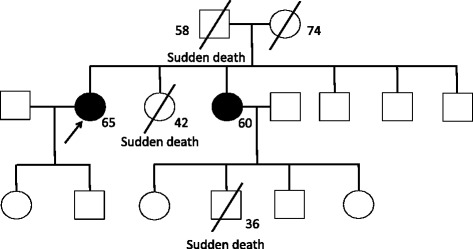
Pedigree of the case. The numbers indicate current age or age at death

**Fig. 2:**
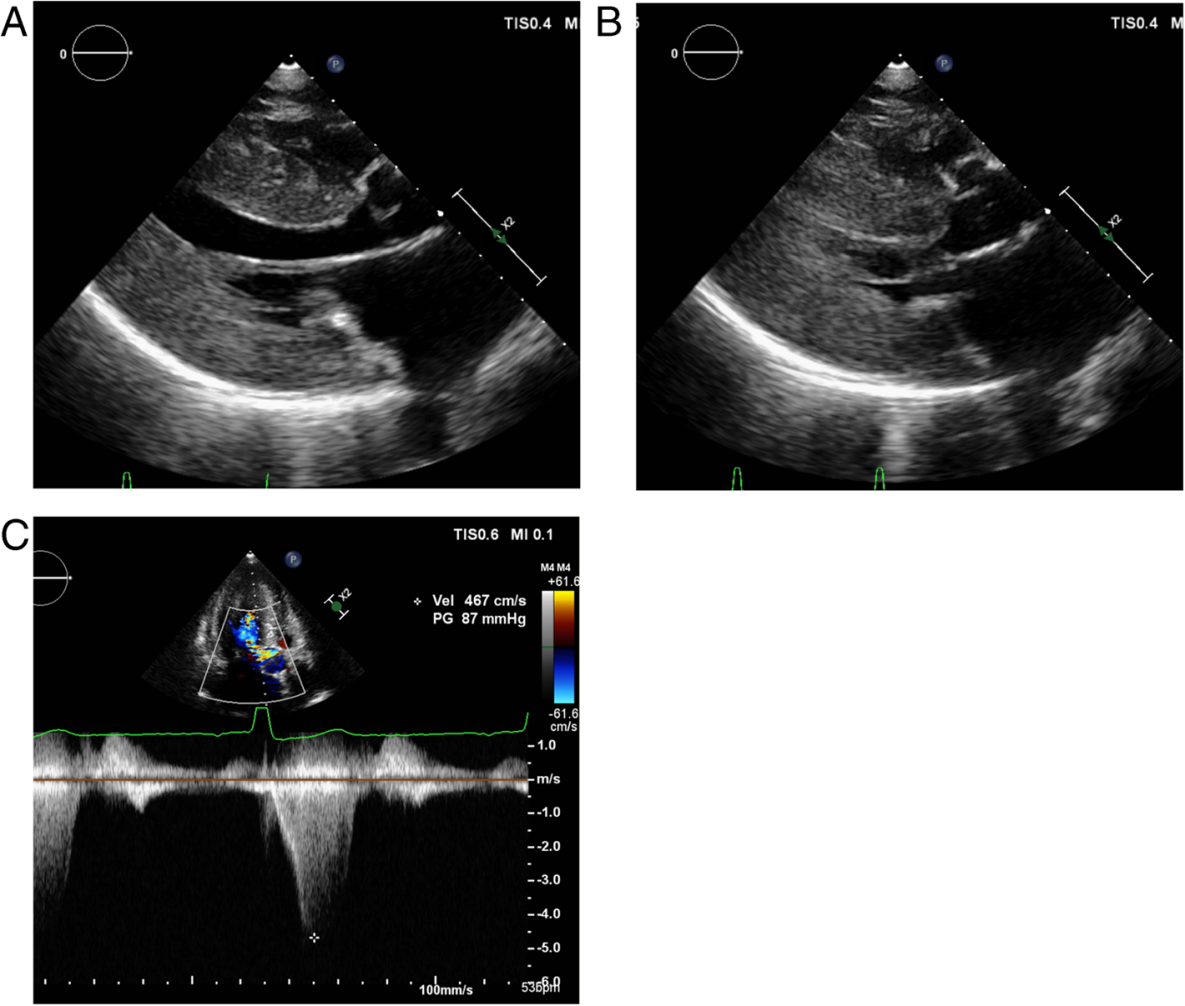
Echocardiographic findings. **a**, **b** Echocardiographic images of para-sternal long axis view of LV showing diffuse cardiac hypertrophy at diastolic (**a**) and systolic (**b**) phases. Systolic anterior motion of anterior mitral leaflet was observed. **c** Measurement of the pressure gradient between mid-LV and LV outflow tract

**Fig. 3:**
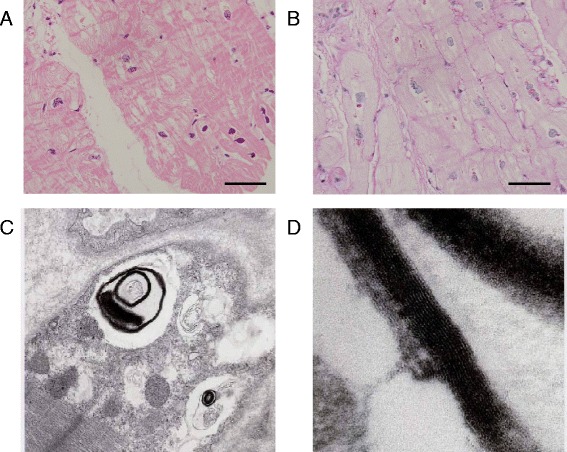
Histological findings of left ventricular endomyocardial biopsy. **a** Vacuolated cardiomyocytes in the left ventricle (hematoxylin and eosin staining). Bar, 50 μm. **b** PAS stain-positive cardiomyocytes with vacuolation. Bar, 50 μm. **c** Zebra body in vacuolated cardiomyocytes. **d** Magnified image of C

**Fig. 4:**
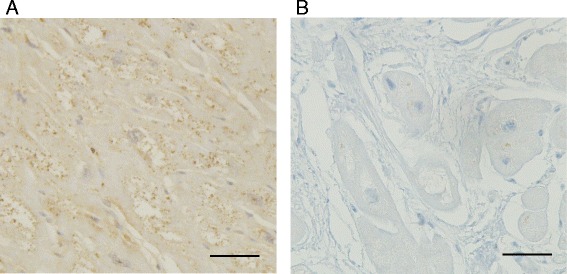
Immunostaining of myocardium against Gb3. **a** Immunostaining of myocardium in typical Fabry disease’s case. Brown staining indicates Gb3 accumulation. Bar, 50 μm. **b** Immunostaining of myocardium in the present case. Bar, 50 μm

A 65-year-old female was referred to our hospital with chest discomfort on effort. On auscultation, a grade III/VI systolic murmur at the 4^th^ left intercostal space was detected. Her blood pressure was 122/66 mmHg. A chest X-ray showed cardiomegaly (cardiothoracic ratio; 0.55) without pulmonary congestion, and an electrocardiogram showed regular sinus rhythm, poor R wave progression in leads V1 to V4, an inverted T wave, and left ventricular (LV) high voltage (SV1 + RV5 = 6.52 mV). She presented no angiokeratoma on her skin, and had no history of acroparesthesia. A medical interview revealed that her father, one of her younger sisters, and a nephew died suddenly at age 58, 42, and 36, respectively (Fig. 1). Another younger sister also presented with LV hypertrophy with outflow obstruction. Transthoracic echocardiography showed diffuse LV hypertrophy with more than 20 mm wall thickness and hyper contraction with LV outflow obstruction. The maximum LV outflow pressure gradient was 87 mmHg, and Valsalva maneuver increased the pressure gradient up to 98 mmHg (Fig. 2a-c). Moderate mitral valve regurgitation was detected due to systolic anterior motion of the anterior mitral leaflet. Laboratory data displayed preserved renal function and no proteinuria. Cardiac catheterization revealed a systolic pressure gradient between mid LV and outflow tract by 82 mmHg, and biopsy specimens presented vacuolation of cardiomyocytes, which were stained by periodic acid-Schiff (PAS) stain (Fig. 3a and b). Zebra bodies were detected by electron microscopic examination in the cells with vacuolation (Fig. 3c and d). Zebra body is a specific finding of Fabry disease, but is also found in other lysosome diseases, such as Niemann-Pick disease, Landing’s disease, Sandhoff’s disease, and mucopolysaccharidosis. The patient had no clinical findings consistent with those diseases. Cationic amphiphilic drugs, including gentamycin, hydroxychloroquine, and amiodarone, are capable of inducing phospholipidosis, leading to deposition of zebra bodies in various cells [4–6], but she had not taken any of these drugs. Although the enzymatic activity of leukocyte GLA was within normal range (62 nmol/mg/h), we initially diagnosed the case with cardiac Fabry disease with the E66Q mutation based on the histological findings. But because the distribution and the density of zebra body were much less than typical cases of Fabry disease, we added the immunostaining against Gb3 in the specimen of LV myocardium. As compared to a case of typical Fabry disease (Fig. 4a), there was few deposition of Gb3, even in vacuolated cells (Fig. 4b). We also analyzed plasma levels of Gb3, but it was within normal range (2.8 μg/ml). In contrast, when we analyzed the case for mutations in the disease genes for HCM [7, 8], it was revealed that the patient carried heterozygous mutations of *MYBPC3* (Gly1009Val) and *MYH6* (Ser624del). Although both mutations were novel, they were not found in the public sequence databases including dbSNP [9], 1000 genomes [10], and Human Genetic Variation database [11]. In addition, the *MYBPC3* mutation was predicted to be a disease-causing mutation by three different in *silico* studies; predicted to be disease-causing by Mutation Taster [12], probably damaging (score 1.000) by PolyPhen-2 [13], and damaging (score 0) by SIFT [14]. As for the *MYH6* mutation, one amino acid at 624th position in the head domain was deleted, which might be deleterious for the function of α-myosin heavy chain. However, because MYH6 is mainly expressed in the atrial muscles and not in the ventricular muscles in human adult hearts, pathological role of the *MYH6* mutation in cardiac hypertrophy in this case might be less significant than the *MYBPC3* mutation. From these observations, HOCM was more reasonable to explain the pathophysiology in the case, although the disease modifying effect of the E66Q mutation cannot be ruled out.

Several reports have already shown that E66Q mutation is accompanied with LV hypertrophy and/or renal dysfunction [15, 16], but histological evidence of Fabry disease has not yet been demonstrated. One report showed typical findings of classic-type Fabry disease with E66Q mutation, but the patient also had another R112C mutation [17]. In the present case, both PAS-positive vacuolated cardiomyocytes and small number of zebra bodies were observed in LV myocardium, but no accumulation of Gb3, indicating that those histological changes were not associated with Fabry disease. A recent study showed that the frequency of the E66Q allele was not different between hemodialysis patients and the general Japanese population [18]. Therefore, thorough genetic analysis is needed to perform differential diagnosis of Fabry disease. Although E66Q is considered as functional polymorphism, not pathogenic mutation, Nakamura et al. showed that the frequency of the E66Q mutation was significantly higher in patients with cerebral small vessel occlusion [19]. Thus, we need to accumulate the clinical data of patients with E66Q mutation in order to elucidate the pathological roles of E66Q mutation.

## Conclusions

This is the confusable case of HOCM with Fabry disease. We have to take into consideration the possibility that some patients with the E66Q mutation may have similar histological findings of Fabry disease, and should be examined the possibility for harboring HCM-related gene mutations.

### Consent

Written informed consent was obtained from the patient for publication of this case report and any accompanying images. A copy of the written consent is available for review by the Editor of this journal. The study was approved by the Ethics Committee of the Fukushima Medical University School of Medicine.

## Abbreviations

Gb3: globotriaosylceramide
GLA: α-galactosidase A
HCM: hypertrophic cardiomyopathy
HOCM: hypertrophic obstructive cardiomyopathy
LV: left ventricle
PAS: periodic acid-Schiff
